# Exploring the Rise of E-cigarette Use Among Male Adolescents in Al-Ahsa, Saudi Arabia: Prevalence, Patterns, and Influencing Factors

**DOI:** 10.7759/cureus.51644

**Published:** 2024-01-04

**Authors:** Muneera Alabdulqader, Mohannad A Almulhim, Mohammed Alquraini, Insaf Ali, Muneera S Alhajri, Noor A Alsaleh, Abdulrahman Al Naim, Khalid I Al Noaim, Rabab A Majzoub, Zainab H Alalawi

**Affiliations:** 1 Pediatrics, King Faisal University, Al-Ahsa, SAU; 2 Paediatrics and Child Health, King Faisal University, Al-Ahsa, SAU; 3 College of Medicine, King Faisal University, Al-Ahsa, SDN; 4 Collage of Medicine, King Faisal University, Al-Ahsa, SAU; 5 College of Medicine, King Faisal University, Al-Ahsa, SAU

**Keywords:** saudi arabia, risk factors, prevalence, adolescents, e-cigarette smoking, electronic cigarettes 'e-cigarettes' vaping' e-smoking, cross-sectional study

## Abstract

Background: The rising prevalence of electronic cigarette (E-cigarette) use among adolescents is a major public health concern. This study investigates the prevalence of E-cigarette use among male adolescents in Al-Ahsa, Saudi Arabia, and explores associated factors.

Methodology: A cross-sectional study was conducted from December 2022 to April 2023, involving 476 male students aged 12 to 19. Data were collected through structured questionnaires, covering sociodemographic information, E-cigarette usage patterns, reasons for use, sources of acquisition, awareness of nicotine content, and perceptions of harm. Statistical analysis was performed using SPSS 25 (IBM Corp., Armonk, NY), with significance set at p < 0.05.

Results: The study revealed a prevalence of 17.4% E-cigarette use among participants, with 12.6% using E-cigarettes exclusively and 4.8% concurrently with traditional tobacco cigarettes. Key findings included initiation as early as age eight, sustained and frequent use, and motivations including peer influence (61.4%), curiosity (31.3%), and flavored options (26.5%). Online platforms (34.9%) and shopping malls (28.9%) were the primary sources of E-cigarette acquisition. Most participants were aware of the nicotine content (84.3%) and believed E-cigarettes were harmful (86.7%). Importantly, 69.9% expressed intentions to quit, with 44.6% planning to do so within 30 days. Significant associations were found between E-cigarette use, education level, and having friends who smoke.

Conclusion: This study highlights the prevalence of E-cigarette use among male adolescents in Al-Ahsa, Saudi Arabia, and identifies peer influence, curiosity, and appealing flavors as driving factors. Targeted prevention and intervention programs, along with regulatory efforts to restrict access, are urgently needed to address this growing public health issue. Increasing awareness of E-cigarette risks and providing cessation support are vital steps towards mitigating E-cigarette use among Saudi male adolescents.

## Introduction

Electronic cigarettes (E-cigarettes) are electronic devices that use battery power to heat a liquid solution, producing an aerosol that can be inhaled and may contain nicotine [1]. The use of E-cigarettes is growing and has become a major public issue [2]. E-cigarettes may deliver a comparable sensation to cigarette smoking by providing taste and inhaling sensations [3]. Despite the misconception that they are less dangerous than tobacco products, E-cigarettes are potentially hazardous to the respiratory system [4].

The usage of E-cigarettes among adolescents has grown significantly since they were first introduced to the market in the early 2000s [5]. E-cigarettes are popular among teenagers and are now the most often-used type of tobacco among adolescents in the United States [6]. From 2017 to 2019, there was a significant rise in the use of electronic cigarettes among teenagers, with the prevalence increasing from 11.7% to 27.5% during this period [7]. Several studies have been carried out on the Saudi Arabian population to investigate the use of E-cigarettes. These studies have revealed that among young adults, the prevalence of E-cigarette use ranges from 25% to 33.5% [8,9].

E-cigarette use has been associated with the development of acute lung injury [10]. In addition to its association with acute lung injury, E-cigarette use has been found to impair memory and executive function in adolescents. Furthermore, a study found that E-cigarettes' nicotine and non-nicotine components caused increased aggressiveness, impulsiveness, attention deficits, and suicidal ideation among adolescents [11].

This research aims to investigate the prevalence of E-cigarette use among male adolescents in Alahsa and the factors associated with E-cigarette usage in this age group. Assessing the prevalence and risk factors for E-cigarette use is crucial for creating effective preventative and intervention methods for dealing with this public health concern. This study's findings may help researchers better understand E-cigarette smoking trends and related characteristics among male students in Alahsa, Saudi Arabia.

## Materials and methods

This cross-sectional study aimed to evaluate E-cigarette smoking patterns and associated characteristics among intermediate and high school male students in Alahsa, Saudi Arabia. The study was conducted over a period of six months, from December 2022 to April 2023. Adolescents aged 12 to 19 attending middle and high schools were the target population. The study focused specifically on male students in middle or high school, with the goal of investigating E-cigarette smoking behaviors in this population. Female students and students who failed to meet the age or educational level were excluded from participating in the study. To maintain data integrity and accuracy, any surveys with unresolved or missing responses have been excluded from the final analysis.

The Richard Geiger equation was used to estimate the proper sample size for the study. The determined sample size was 385 people, with a 5% margin of error, a 95% confidence level, and a population size of 1270. A 50% response distribution was assumed to allow for any non-response or incomplete questionnaires. This sample size was designed to give enough statistical power to identify meaningful relationships and reliably estimate prevalence rates.

A standardized paper-based questionnaire was developed to collect data. Regarding the validity of the questionnaire, the research team had a group of experts in pediatrics, biostatistics, and climate science review the questionnaire. The experts were asked to provide feedback on the relevance and accuracy of the questions, as well as their clarity and comprehensibility. Based on the feedback from the experts, the research team made revisions to the questionnaire to ensure that it was measuring the intended constructs accurately and that the questions were appropriate for the target population. The reliability of the questionnaire was assessed by calculating Cronbach's alpha, which was found to be 0.817. This indicates that the items within the questionnaire were highly interrelated and consistent in measuring the underlying construct. The questionnaire's questions were divided into three categories: sociodemographic data (educational level, nationality, academic performance, father’s educational level, mother’s educational level, parent’s and friend’s smoking status), information about E-cigarette smoking habits (frequency, reasons for smoking, and sources of information), and E-cigarette-related questions (flavors, personal motivations for usage, addiction, and attempts to quit vaping).

The research was submitted and approved by the King Faisal University - Ethics Committee in Alahsa, Saudi Arabia (KFU-REC-2022-DEC-ETHICS71). To protect participant privacy, the research protocol ensured complete confidentiality. No identifying data was collected, and the questionnaire was distributed anonymously. Participants were assured that their responses would be kept confidential and used solely for research purposes.

Statistical analysis

The data were collected, reviewed, and added to the Statistical Package for Social Sciences (SPSS) 25 version (IBM Corp., Armonk, NY). All statistical methods were two-tailed with an alpha level of 0.05, considering significance if the P-value is less than 0.05. Descriptive analysis was done by prescribing frequency distribution and percentage for study variables. A cross-tabulation was used to show the association between participant demographics and using E-cigarettes, using the Pearson chi-square test for significance. The subjects were involved in the study after giving informed consent.

## Results

Out of the 476 adolescent males who answered the questionnaire, 4 participants were excluded due to the exclusive smoking of traditional cigarettes. The remaining 472 participants, aged 12 to 19 years (M = 15.76, SD = 1.758), were included in the study. The majority of the participants were non-smokers (82.4%). Among E-cigarette smokers, most were high school students (95.2%), with only a small percentage being non-Saudi (4.8%). The majority of participants reported excellent academic performance (56.6%). Among non-smokers, the majority were high school students (56%), with a significant percentage being Saudi (88.2%). There was no significant difference between smokers' and non-smokers' parents in terms of education level. Most fathers of both E-cigarette smokers (81.9%) and non-smokers (80.7%) were employed. Regarding daily pocket money, a significant proportion of both E-cigarette smokers (33.7%) and non-smokers (48.6%) received an amount between 5 and 10 SAR. Among E-cigarette smokers, 25.3% had a smoking father, while only 1.2% had a smoking mother. Most E-cigarette users (92.8%) had smoking friends. Among non-smokers, 83.3% had a non-smoking father. A significant proportion (72.8%) of non-smokers reported not having smoking friends (Table 1).

**Table 1:** Comparison of different demographic variables between E-cigarette smokers and non-smokers *P < 0.05 (significant)

Variables	N = 472				p-value
	E-cigarettes smokers (n = 83)		Non-smokers (n = 389)		
	n	%	n	%	
Educational level					
Intermediate school	4	4.8%	171	44%	<0.001*
High school	79	95.2%	218	56%	
Nationality					
Saudi	79	95.2%	343	88.2%	0.092
Non-Saudi	4	4.8%	46	11.8%	
Academic performance					
Excellent	47	56.6%	241	62%	0.503
Very good	30	36.1%	115	29.6%	
Good	6	7.2%	28	7.2%	
Below average	0	0%	5	1.3%	
Father’s educational level					
Primary school	6	7.2%	24	6.2%	0.387
Intermediate school	3	3.6%	41	10.5%	
High school	27	32.5%	124	31.9%	
College	27	32.5%	109	28%	
Post-graduate	20	24.1%	91	23.4%	
Mother’s educational level					
Primary school	4	4.8%	31	8%	0.893
Intermediate school	9	10.8%	39	10%	
High school	22	26.5%	100	25.7%	
College	34	41%	150	38.6%	
Post-graduate	14	16.9%	69	17.7%	
Does the father work?					
Yes	68	81.9%	314	80.7%	0.920
No	15	18.1%	75	19.3%	
Does the mother work?					
Yes	35	42.2%	136	35%	0.265
No	48	57.8%	253	65%	
Daily pocket money					
Less than 5 SAR	10	12%	51	13.1%	0.055
5-10 SAR	28	33.7%	189	48.6%	
10-15 SAR	23	27.7%	74	19%	
More than 15 SAR	22	26.5%	75	19.3%	
Is the father a smoker?					
Yes	21	25.3%	65	16.7%	0.092
No	62	74.7%	324	83.3%	
Is the mother a smoker?					
Yes	1	1.2%	0	0	0.394
No	82	98.8%	389	100%	
Do you have any friends who smoke?					
Yes	77	92.8%	106	27.2%	<0.001*
No	6	7.2%	283	72.8%	

A chi-square test was employed to investigate the association between various factors and the use of E-cigarettes among a cohort of 472 participants. The results unveiled significant correlations between E-cigarette usage and two key variables: education level and the presence of friends who smoke. In the E-cigarette smoking group, a substantially higher proportion of high school participants (95.2%) was observed compared to the non-smoking group (56%), with a p-value of <0.001, signifying a strong association. Likewise, within the E-cigarette smoking group, a significantly greater percentage of participants who had friends who smoked (92.8%) was noted, in contrast to only 27.2% in the non-smoking group, and this difference also yielded a p-value of <0.001. Conversely, no statistically significant relationships were identified between E-cigarette usage and factors such as nationality, academic performance, parental education, smoking status, and daily pocket money, highlighting their lack of association with E-cigarette smoking (Table 1).

The dataset examined electronic cigarette (E-cigarette) usage patterns among 83 participants who reported having used E-cigarettes. Among this cohort, participants' initial experimentation with E-cigarettes commenced at varying ages, ranging from as early as eight years old to as late as 18 years old. The mean age at which participants first tried e-cigarettes was found to be 14.55 years, with a standard deviation of 2.074. The majority of participants, constituting 26.5% and 27.7%, respectively, initiated E-cigarette use at the ages of 14 and 15. A smaller proportion of participants reported trying E-cigarettes at ages 16 (18.1%), 13 (7.2%), 17 (8.4%), 10 (1.2%), 11 (2.4%), and 18 (3.6%) years old. Notably, only a small segment of the sample, comprising 4.8%, indicated that they had tried E-cigarettes at a remarkably young age of eight or younger. No participants within the sample reported experimenting with E-cigarettes at ages 9, 12, or 19 years or older. Concerning the frequency of E-cigarette usage throughout their lifetime, a substantial number of participants (51.8%) revealed that they had employed E-cigarettes for more than 100 days. Conversely, 10% of respondents (10.8%) reported limited use, specifically less than ten days. A diverse distribution emerged considering the usage frequency within the past 30 days. A noteworthy proportion of participants (22.9%) indicated daily E-cigarette use. Additionally, other usage patterns included 1-2 days (16.9%), 3-5 days (19.3%), 6-9 days (10.8%), 10-19 days (12%), and 20-29 days (18.1%). These findings provide a comprehensive overview of the diverse usage patterns exhibited by the participants within the specified time frames (Table 2).

**Table 2:** E-cigarette smoking behavior and habits among male adolescents

Questions	Frequency	Percentage
Days of using electronic cigarettes in the entire life		
One day	8	9.6%
2–10 days	1	1.2%
11–20 days	4	4.8%
21–50 days	10	12%
51–100 days	17	20.5%
More than 100 days	43	51.8%
Days of using electronic cigarettes in the past 30 days		
1–2 days	14	16.9%
3–5 days	16	19.3%
6–9 days	9	10.8%
10–19 days	10	12%
20–29 days	15	18.1%
Everyday	19	22.9%
What are your reasons for using electronic cigarettes?		
A friend uses it	51	61.4%
I am curious about it.	26	31.3%
I can use it without anyone noticing it at home or school	24	28.9%
It is available in flavors such as mint, candy, fruit, or chocolate	22	26.5%
I have seen people using it on TV, internet, or in movies	12	14.5%
A family member uses it	10	12%
It is less harmful than other forms of tobacco	9	10.8%
Trying to quit using other tobacco products	7	8.4%
It is easier to obtain than other tobacco products	5	6%
It costs less than other tobacco products	5	6%
During the past 30 days, from where did you get or buy the electronic cigarettes?		
Online	29	34.9%
From a friend	29	34.9%
Shopping mall	24	28.9%
Grocery store	19	22.9%
Gas station or small store	14	16.9%
From a family member	4	4.8%
From someone else who is not a family member or friend	2	2.3%
Other	1	1.1%
Are you aware that electronic cigarettes contain nicotine?		
Yes	70	84.3%
No	11	13.3%
Not sure	2	2.4%
Do you think that electronic cigarettes are harmful to health?		
Yes	72	86.7%
No	8	9.6%
Not sure	3	3.6%
Are you seriously considering quitting electronic cigarettes?		
Yes, in the coming 30 days	37	44.6%
Yes, in the coming 6 months	8	9.6%
Yes, in the coming 12 months	13	15.7%
Yes, but not in the coming 12 months	8	9.6%
No	17	20.5%
During the past 12 months, how many times have you stopped using electronic cigarettes for one day or more because you were trying to quit using them permanently?		
I haven’t tried it during the past 12 months	26	31.3%
One time	28	33.7%
2 times	24	28.9%
3–5 times	0	0%
6–9 times	1	1.2%
10 times or more	4	4.8%

It is important to note that participants had the option to select multiple reasons for their E-cigarette use. The predominant and most frequently cited rationale for E-cigarette utilization was peer influence, with a substantial majority of 61.4% (51 participants) indicating that they had adopted e-cigarettes due to the influence of friends who also engaged in E-cigarette use. Furthermore, curiosity emerged as a compelling factor, with 31.3% (26 participants) disclosing that they had tried E-cigarettes primarily out of curiosity. The appeal of a diverse range of flavors, including mint, candy, fruit, or chocolate, was another noteworthy motive, cited by 26.5% (22 participants) of users. A significant proportion of participants (28.9%, 24 individuals) acknowledged using E-cigarettes as they provided a discreet option for consumption, allowing them to go unnoticed at home and in school. It is also noteworthy that a smaller segment of participants reported employing E-cigarettes as a means to either quit using other tobacco products, such as traditional cigarettes (8.4%, 7 participants), or because they perceived E-cigarettes as a less harmful alternative to conventional tobacco products (10.8%, 9 participants). Media influence played a role for some respondents, as 14.5% (12 participants) indicated that they had initiated E-cigarette use after witnessing its portrayal in television programs, internet content, or movies. In comparison, a subset of participants (6%, five individuals) reported choosing E-cigarettes due to their perceived cost-effectiveness compared to other tobacco products, such as cigarettes, while an equal proportion (6%, 5 participants) cited the ease of accessibility as a motivating factor (Table 2).

Online platforms were indicated as the predominant source for getting E-cigarettes by 34.9% of participants (29 people), followed by shopping malls (28.9%, 24 people) and grocery shops (22.9%, 19 people). Gas stations and smaller convenience stores provided services to 16.9% of participants (14 people). The interpersonal exchange was noticeable, with 34.9% of participants (29 people) acquiring E-cigarettes from friends and just a small fraction (2.4%) receiving them from non-friends or family members. A minor proportion of participants disclosed that they sourced E-cigarettes within their own families, constituting 4.8% of the sample. Furthermore, a solitary participant (1.2%) indicated an alternative, unspecified source categorized as "other." We found that most E-cigarette smokers were aware that E-cigarettes contain nicotine (70 participants, 84.3%). Regarding the perceptions of the harm of E-cigarettes, most participants (72 participants, 86.7%) reported that they believed E-cigarettes were harmful to health (Table 2).

Most participants (58 participants, 69.9%) seriously considered quitting E-cigarettes. Among those who reported considering quitting, the most significant proportion (37 participants, 44.6%) reported that they intended to quit in the coming 30 days, while 8 participants (9.6%) planned to quit in the coming six months, and 13 participants (15.7%) intended to quit in the coming 12 months. Some participants (8 participants, 9.6%) reported that they intended to quit but not within the coming 12 months, while 17 participants (20.5%) reported that they had no intention of quitting. Regarding past attempts to quit E-cigarettes, the largest proportion of participants reported trying to quit one time during the past 12 months (28 participants, 33.7%), followed by two times (24 participants, 28.9%), and ten times or more (4 participants, 4.8%). Some participants (26 participants, 31.3%) reported not trying to quit E-cigarettes during the past 12 months, while only 1 participant (1.2%) reported trying to quit 6-9 times during the past 12 months (Table 2).

## Discussion

Our study investigated smoking behavior and exposure among adolescents in Saudi Arabia, specifically focusing on using E-cigarettes. The study included 472 participants aged 12 to 19 years, with an average age of 15.76 years. There was a prevalence of 17.6%, and most participants were high school students, which reflects the importance of targeting this age group for interventions and preventive measures. Notably, most participants were Saudi, which may indicate a need for culturally sensitive approaches to addressing smoking behavior in this population.

When examining the participants' smoking behavior, it became evident that the majority of the sample did not engage in the use of either E-cigarettes or traditional tobacco cigarettes. This indicates a relatively low prevalence of smoking within the studied population, with only 17.6% of participants reporting E-cigarette use. This is inconsistent with what has been found in a previous study by Goniewicz et al., which reported that 30% of the youth population reported using E-cigarettes. Children who smoke tend to conceal their habits from their parents. For this reason, the prevalence of E-cigarette usage among children was found to be 13.3%, according to an earlier study, which was predicted to be underestimated. Because of this, it is difficult to estimate the exact number of E-cigarette users [12].

The study also emphasized the usage patterns of E-cigarettes among participants who reported using them. The age at which participants first tried E-cigarettes ranged from 8 to 18 years old, with the majority trying them at 14 or 15 years old. Thomas et al. reported a similar finding, reporting that the average age of first E-cigarette use was around 14 years old. Nevertheless, many teenagers claimed to have started using electronic cigarettes even earlier [13]. It is concerning that a small proportion of participants (4.8%) reported trying E-cigarettes at the age of eight or younger, indicating the potential accessibility of these products to underage individuals.

Furthermore, the study assessed the frequency of E-cigarette use in terms of days used in participants’ lives and the past 30 days. It was found that a significant proportion of participants (51.8%) reported using E-cigarettes for more than 100 days in their entire lives, indicating sustained use. Moreover, 22.9% of participants reported using E-cigarettes daily in the past 30 days, suggesting frequent and regular use among a subset of users. The sustained and frequent use is aligned with a study by Vogel et al., which also reported that a subgroup of adolescents exhibited regular and continuous E-cigarette use [14]. According to a different survey, many high school and middle school students who use E-cigarettes reported using them regularly during a 30-day period. Notably, 17.2% of middle school students and 43.6% of high school students said they had used E-cigarettes for 20 or more days in the previous 30 days. In addition, 8.3% of current middle school users and 27.6% of current high school users reported using E-cigarettes daily [15]. E-cigarette usage among teenagers may become a habit, as evidenced by the fact that more than half of the study's participants said they had used them for more than 100 days throughout their lives. The high percentage of daily users during the previous 30 days further raises concerns about addiction and the possibility of long-term damage.

Regarding the reasons for using E-cigarettes, the most commonly reported motivation was peer influence, with a significant proportion of participants (61.4%) reporting that they used E-cigarettes because their friends used them. This finding is consistent with previous research, which also highlighted the influence of peer networks on E-cigarette use among adolescents, and it was shown that teenagers who start vaping or who already do so are more likely to continue vaping if their peers also vape or if they find new friends who do as well [16]. Curiosity emerged as another prominent reason for using E-cigarettes, with nearly one-third of participants (31.3%) indicating they were curious about E-cigarettes. This finding aligns with previous studies that have identified curiosity as a significant motivating factor for E-cigarette experimentation among youth [17]. Furthermore, a substantial number of participants reported the availability of appealing flavors (26.5%). Flavors such as mint, candy, fruit, or chocolate have been recognized as a critical marketing strategy for E-cigarette manufacturers to attract young users [18]. The pleasant taste and variety of flavors might enhance adolescents' appeal and perceived enjoyment of E-cigarette use. The ability to use E-cigarettes discreetly without detection at home or school was also identified as a motivation for use by a considerable proportion of participants (28.9%). This finding suggests that the discreet nature of E-cigarettes, including their design and lack of noticeable odor, might facilitate surreptitious use among youth [19].

Interestingly, a small proportion of participants reported using E-cigarettes to quit using other tobacco products (8.4%) or because they believed E-cigarettes were less harmful than other forms of tobacco (10.8%). These findings are compatible with prior research highlighting the use of E-cigarettes as a harm reduction or smoking cessation tool among specific individuals [20]. However, it is worth noting that the effectiveness of E-cigarettes as a cessation aid and their long-term health implications remain a topic of debate and require further investigation [21].

Turning to the sources of obtaining E-cigarettes, online platforms emerged as the most commonly reported source among the participants (34.9%). This finding is consistent with the increasing prevalence of online E-cigarette sales and the ease of access provided by digital platforms [22]. Regulatory efforts to curb online sales and enforce age restrictions may be necessary to mitigate underage access to E-cigarettes. Shopping malls were also reported as a significant source of E-cigarettes (28.9%). This finding suggests that the availability and accessibility of E-cigarettes in retail settings, such as malls, contribute to their widespread use among adolescents [23]. Implementing stricter regulations and age verification measures for retailers selling E-cigarettes may help limit underage access.

Awareness of nicotine content in E-cigarettes was relatively high among the participants, with the majority (84.3%) reporting awareness. In contrast to our survey, another study discovered that 20.4% of young people who tried E-cigarettes said they were unaware if their first E-cigarette had any nicotine [24]. Despite high awareness, a small proportion of participants remained unaware (13.3%) or uncertain (2.4%) of the presence of nicotine in E-cigarettes. This finding highlights the need for continued education and public health campaigns targeting youth to enhance their understanding of the potential risks associated with nicotine use in E-cigarettes [25].

Concerning perceptions of the harm of E-cigarettes, most participants (86.7%) believed that E-cigarettes were harmful to health. Compared to our findings, a previous study revealed that fewer than half of the participants believed E-cigarettes threatened their health [26]. This finding aligns with growing evidence highlighting the potential risks associated with E-cigarette use, including nicotine addiction, respiratory issues, and cardiovascular effects [27]. However, it is worth noting that a small proportion of participants (9.6%) did not believe that E-cigarettes were harmful. This discrepancy in perceptions may stem from varying levels of awareness, misinformation, or conflicting messages regarding the safety of E-cigarettes in certain contexts [28]. These findings reinforce the importance of targeted education campaigns to increase awareness of the potential risks associated with E-cigarette use.

Examining the participants' intentions and past attempts to quit E-cigarettes, a significant proportion reported considering quitting (69.9%), demonstrating a readiness for change. Furthermore, among those who intended to quit, a substantial proportion aimed to do so within the next 30 days (44.6%), suggesting a sense of urgency and motivation to discontinue E-cigarette use. Regarding past attempts to quit, a noteworthy number of participants reported making one or multiple quit attempts within the past 12 months. Cuccia et al. found that almost one-third of young people using E-cigarettes have tried to stop using them in the last year. Furthermore, 15% of participants said they intended to quit using E-cigarettes within the next 30 days, while 54% of participants said they planned to stop using them in the future [29]. This finding underscores the importance of addressing cessation strategies tailored specifically to E-cigarette users, particularly among young individuals.

The results revealed that education level and having friends who smoke were significantly associated with E-cigarette smoking. A meta-analysis found a strong positive correlation between adolescent E-cigarette usage and the use of E-cigarettes by friends and family [30]. However, it is important to note that no significant relationships were found between nationality, academic performance, parental education, daily pocket money, and E-cigarette smoking. These results may indicate that E-cigarette use is influenced by a complex interplay of various factors beyond those examined in this study, such as individual attitudes, personal experiences, and the broader sociocultural context.

Limitations and implications

The study has several limitations, including using a cross-sectional design, which precludes causal inferences, and relying on self-reported data, which may be subject to recall and social desirability biases. Additionally, the study was limited to a single city, which may limit the generalizability of the findings to other regions in Saudi Arabia. Moreover, we did not specify whether the students attended public or private schools in the questionnaire, which may reflect the students' socioeconomic status and the school's policies.

Nevertheless, the study has important implications for public health interventions to prevent and reduce E-cigarette use among adolescents in Saudi Arabia. The findings highlight the need for targeted and culturally appropriate prevention programs that address the influence of peer networks and the availability of E-cigarettes online.

## Conclusions

In conclusion, the present study sheds light on the prevalence, usage patterns, sources, and perceptions of E-cigarettes among adolescents in Saudi Arabia. The study found that a small proportion of participants reported using E-cigarettes, primarily due to peer influence, and that they were aware of the nicotine content in E-cigarettes and believed E-cigarettes to be harmful to health. Furthermore, the study found that the most common source of buying E-cigarettes was online and that most E-cigarette users intended to quit or had attempted to quit E-cigarettes in the past. The study also found that education level and having friends who smoke were significantly associated with trying E-cigarettes, highlighting the importance of targeted prevention strategies.

## Appendices

Appendix 1

**Table 3:** The questionnaire regarding E-cigarette use in male teenagers in Al-Ahsa, Saudi Arabia

Questions	Choose your answer
Q1: Gender	1. Male
	2. Female
Q2: Educational level	1. Intermediate school
	2. High school
Q3: Age	1. 12
	2. 13
	3. 14
	4. 15
	5. 16
	6. 17
	7. 18
	8. 19 or older
Q4: What level are you in?	1. Level 1
	2. Lever 2
	3. Level 3
Q5: Nationality	1. Saudi
	2. Non-Saudi
Q6: Academic performance	1. Excellent
	2. Very good
	3. Good
	4. Below average
Q7: Father’s level of education	1. Primary school
	2. Intermediate school
	3. High school
	4. College
	5. Postgraduate
Q8: Mother’s level of education	1. Primary school
	2. Intermediate school
	3. High school
	4. College
	5. Postgraduate
Q9: Does the father work?	1. Yes
	2. No
Q10: Does the mother work?	1. Yes
	2. No
Q11: How much is the daily pocket money allowance in SAR?	1. Less than 5 SAR
	2. 5-10 SAR
	3. 10-15 SAR
	4. More than 15 SAR
Q12: Is the father a smoker?	1. Yes
	2. No
Q13: Is the mother a smoker?	1- yes
	2- no
Q14: Do you have any friends who smoke?	1. Yes
	2. No
Q15: Do you use E-cigarettes or traditional cigarettes for smoking?	1. E-cigarettes
	2. Traditional cigarettes
	3. Both
	4. I don’t use any of them
Q16: How old were you when you first used an electronic cigarette?	1. 8 or younger
	2. 9
	3. 10
	4. 11
	5. 12
	6. 13
	7. 14
	8. 15
	9. 16
	10. 17
	11. 18
	12. 19 or older
Q17: What are your reasons for using electronic cigarettes? (choose all that apply)	1. A friend uses it
	2. A family member uses it
	3. Trying to quit using other tobacco products such as cigarettes
	4. It costs less than other tobacco products such as cigarettes
	5. It is easier to obtain than other tobacco products such as cigarettes
	6. I have seen people using it on TV, internet, or in movies
	7. It is less harmful than other forms of tobacco such as cigarettes
	8. It is available in flavors such as mint, candy, fruit, or chocolate
	9. I can use it without anyone noticing it at home or school
	10. I am curious about it
	11. Other
Q18: Overall, approximately how many days have you used electronic cigarettes in your entire life?	1. One day
	2. 2–10 days
	3. 11–20 days
	4. 21–50 days
	5. 51–100 days
	6. More than 100 days
Q19: During the past 30 days, how many days have you used electronic cigarettes?	1. 1–2 days
	2. 3–5 days
	3. 6–9 days
	4. 10–19 days
	5. 20–29 days
	6. Every day
Q20: During the past 30 days, from where did you get or buy the electronic cigarettes? (choose all that apply)	1. Gas station or small store
	2. Grocery store
	3. Shopping mall
	4. Online
	5. From a family member
	6. From a friend
	7. From someone else who is not a family member or friend
	8. Other
Q21: Are you aware that electronic cigarettes contain nicotine?	1. Yes
	2. No
	3. Not sure
Q22: Do you think that electronic cigarettes are harmful to health?	1. Yes
	2. No
	3. Not sure
Q23: Are you seriously considering quitting electronic cigarettes?	1. Yes, in the coming 30 days
	2. Yes, in the coming six months
	3. Yes, in the coming 12 months
	4. Yes, but not in the coming 12 months
	5. No
Q24: During the past 12 months, how often have you stopped using electronic cigarettes for one day or more because you were trying to quit using them permanently?	1. I didn’t try during the past 12 months
	2. One time
	3. 2 times
	4. 3-5 times
	5. 6-9 times
	6. 10 times or more
